# Precision Microbiome Modulation: Exploring *Lactobacillus* spp. as A Targeted Strategy for Type 2 Diabetes Management

**DOI:** 10.1007/s13668-026-00739-3

**Published:** 2026-03-04

**Authors:** Ann Sze Cheah, Ji Wei Tan, Faizul Jaafar, Wendy Wai Yeng Yeo

**Affiliations:** 1 School of Science, Monash University Malaysia, Jalan Lagoon Selatan, 47500 Bandar Sunway, Selangor Darul Ehsan Malaysia; 2 Department of Biochemistry, Faculty of Medicine, Universiti Kebangsaan Malaysia, 56000 Cheras, Kuala Lumpur Malaysia; 3 School of Pharmacy, Monash University Malaysia, Jalan Lagoon Selatan, 47500 Bandar Sunway, Selangor Darul Ehsan Malaysia

**Keywords:** Lactobacillus, Type 2 diabetes mellitus, Probiotics, Gut microbiota, Precision medicine, Pharmacomicrobiomics

## Abstract

**Purpose of Review:**

The International Diabetes Federation projects a prevalence of 783.2 million cases of type 2 diabetes mellitus (T2DM) by 2045. The escalation in healthcare expenditure and adverse effects of current drugs necessitate a clinically effective and low-cost alternative therapeutic option with minimal health complications. *Lactobacillus* spp., a well-studied gut probiotic has shown promising antidiabetic effects against T2DM in several randomized controlled trials. However, the exact mechanisms of *Lactobacillus* spp. in regulating the glycaemic profile are not well elucidated, limiting the optimization of probiotic-based interventions. Therefore, this paper aims to explore the various antidiabetic mechanisms of *Lactobacillus* spp., with a focus on their integration into precision medicine approaches.

**Recent Findings:**

*Lactobacillus* spp. supplementation alleviates insulin resistance in T2DM by modulating glucose metabolism and transport, exerting anti-inflammatory and anti-oxidative effects as well as restructuring gut microbiota. *Lactobacillus* spp. also enhance the functional properties of food by increasing the antioxidant capacity and improving glucose metabolism. Despite these promising effects, clinical translation is challenged by strain-specific survivability in the gastrointestinal tract and safety concerns. Thus, leveraging precision medicine via tailoring treatments based on individual microbiome profiles, genetic backgrounds and metabolic phenotype may unravel the full therapeutic potential of targeted *Lactobacillus* spp. for T2DM treatment. This approach can be reached by integrating multi-omics profiling and artificial intelligence technologies.

**Summary:**

Lactobacillus spp. may act as adjunctive support in the management T2DM rather than stand-alone therapies in T2DM management, as their efficacy is dependent on appropriate dietary interventions and metabolic context. Altogether, integrating Lactobacillus spp. into personalized treatment frameworks offers a promising avenue for developing more targeted, effective and safe interventions for T2DM.

## Introduction

Type 2 diabetes mellitus (T2DM) is a metabolic disorder that manifests as hyperglycaemia, arising from insulin resistance and impaired pancreatic beta cell function [1]. Common risk factors for T2DM include genetic predisposition, a sedentary lifestyle and high-fat diets [2]. Poor management of T2DM results in persistent hyperglycaemia and leads to various adverse complications such as neuropathy, nephropathy and retinopathy [3]. Furthermore, uncontrolled hyperglycaemia weakens the immune system, making diabetic patients more prone to infections such as skin, urinary tract, respiratory and diabetic foot infections, often caused by pathogens such as *E. coli* and *Klebsiella* [4]. Studies revealed that inflammatory response plays a pivotal role in the development of insulin resistance, a hallmark of T2DM [5]. It is suggested that inflammation triggers the production of pro-inflammatory molecules including cytokines and adipocytokines through various pathways, including oxidative and metabolic stress which interfere with insulin signalling pathways, exacerbating insulin resistance and hyperglycaemia [5, 6].

The International Diabetes Federation estimated a global diabetes prevalence of 536.6 million in 2021 and an expected 46% increase to 783.2 million in 2045, with middle-income countries being the most affected, attributing to substantial population growth [7]. Additionally, the global healthcare expenditure for the management of diabetes and its associated complications, projected to surpass one trillion USD by 2045, poses a substantial financial burden especially to individuals from low-middle income countries which account for approximately 80% of the patient population [7]. The anticipated prominent rise in T2DM prevalence in middle-income countries and concomitant economic burden manifest the need for an alternative treatment. The inter-individual variability in drug response can implicate adverse drug reactions, paradoxically necessitating further prolonged treatment and imposing significant health and economic burdens [8].

Currently, various drug options with different mechanisms of action are available for T2DM treatment. Insulin sensitizers, including biguanides and thiazolidinediones, promote insulin sensitivity, whereas both insulin secretagogues, such as sulfonylureas and meglitinides, and incretin-based therapy, such as dipeptidyl peptidase-IV (DPP-IV) inhibitors and glucagon-like peptide-1 (GLP-1) receptor agonists, stimulate insulin production [9]. Furthermore, α-glucosidase inhibitors reduce intestinal glucose absorption, whereas sodium-glucose cotransporter-2 (SGLT2) inhibitors inhibit renal glucose reabsorption [9]. However, these pharmaceutical agents have low efficacy, limited tolerability and are associated with gastrointestinal symptoms such as diarrhoea and vomiting, weight gain as well as hypoglycaemia [10].

Recent advances in genomics have ushered in a new era of precision medicine, which tailors disease prevention and treatment based on an individual’s genomic profile, lifestyle and environmental exposures [11]. One area of growing interests in the search for alternative treatment for T2DM is the emergence of pharmacomicrobiomics, which explores the influence of human microbiome variation in drug response and disposition that provides insights into the complex host-environment-human microbiome interactions [12–14]. In this context, probiotics such as *Lactobacillus* spp. represent a promising avenue for precision microbiome modulation to improve glycaemic control and metabolic outcomes in T2DM.

The gut microbiota is crucial in modulating host metabolism. Thus, gut dysbiosis is implicated in the pathogenesis of T2DM and other metabolic disorders as it significantly influences the regulation of inflammation, energy homeostasis, gut barrier function, glucose and lipid metabolism, insulin sensitivity as well as dietary interactions [15]. Dysbiosis is defined as the compositional imbalance of resident commensal communities, alterations in bacterial metabolic activities, or shifting of the bacterial distribution within the gut [16].The T2DM microbiota profile is characterized by an increased relative abundance of opportunistic pathogens and a decreased relative abundance of butyrate-producing bacteria, which further signifies the link between gut dysbiosis and T2DM development [17]. This paradigm shift has generated a new perspective in T2DM treatment: probiotics as a novel therapeutic alternative for a more nuanced approach to treatment through precision medicine.

Probiotics are live bacteria that benefit the host’s health when administered sufficiently [18] Unlike conventional drug regimens, probiotics are generally safer, non-invasive, more accessible, inexpensive and associated with fewer side effects [19–21]. Long-term probiotic administration also establishes a stable colonization to provide an enduring effect [22]. Recent meta-analysis has corroborated the positive role of probiotics in glycaemic control in human trials, as supported by the clinically significant decrease in fasting insulin, fasting blood glucose (FBG), and homeostasis model of assessment of insulin resistance [22, 23]. Among the probiotics, *Lactobacillus* species are the most studied and widely commercialized [24]. Members of the *Lactobacillus* genus are vital probiotic bacteria in the gut microbiome and have a prominent role as a therapeutic approach for liver diseases and cardiovascular-related diseases in clinical studies [25–27].

Multiple randomized placebo-controlled trials have demonstrated the clinical effectiveness of *Lactobacillus* spp. in regulating the glycaemic profile in T2DM patients [1, 28–31]. However, the mechanisms involved have not been defined clearly, which impedes probiotic therapy development. Therefore, this review aims to investigate the antidiabetic mechanisms of *Lactobacillus* spp. as a targeted and personalized strategy to treat T2DM. Additionally, this paper will evaluate the potential challenges associated with *Lactobacillus* spp. supplementation and suggest methods to improve the application feasibility.

## Research Methodology

This narrative review was carried out with a structured search strategy, though not fully systematic. We conducted searches in several scientific databases, including PubMed, Scopus and Web of Science, along with other relevant sources from database inception to 1 June 2024, with the final search conducted on 1 December 2025. The search terms included key concepts related to probiotics and diabetes, such as “Lactobacillus,” “probiotics,” “gut microbiota,” “type 2 diabetes mellitus,” “insulin resistance,” “fermented foods,” and “precision medicine.”

We included original research articles involving human or animal models that met the following criteria: (i) the study investigated *Lactobacillus* spp. or products enriched with *Lactobacillus* as the primary intervention or exposure; and (ii) it reported outcomes related to glycemic control, insulin resistance or gut microbiota composition in the context of T2DM. Both clinical trials and *in vivo* studies were considered. Only studies published in English were included. Editorials, conference abstracts and studies not involving *Lactobacillus* or not relevant to T2DM-related outcomes were excluded.

## Targeted *Lactobacillus* spp. In T2DM

**Fig. 1:**
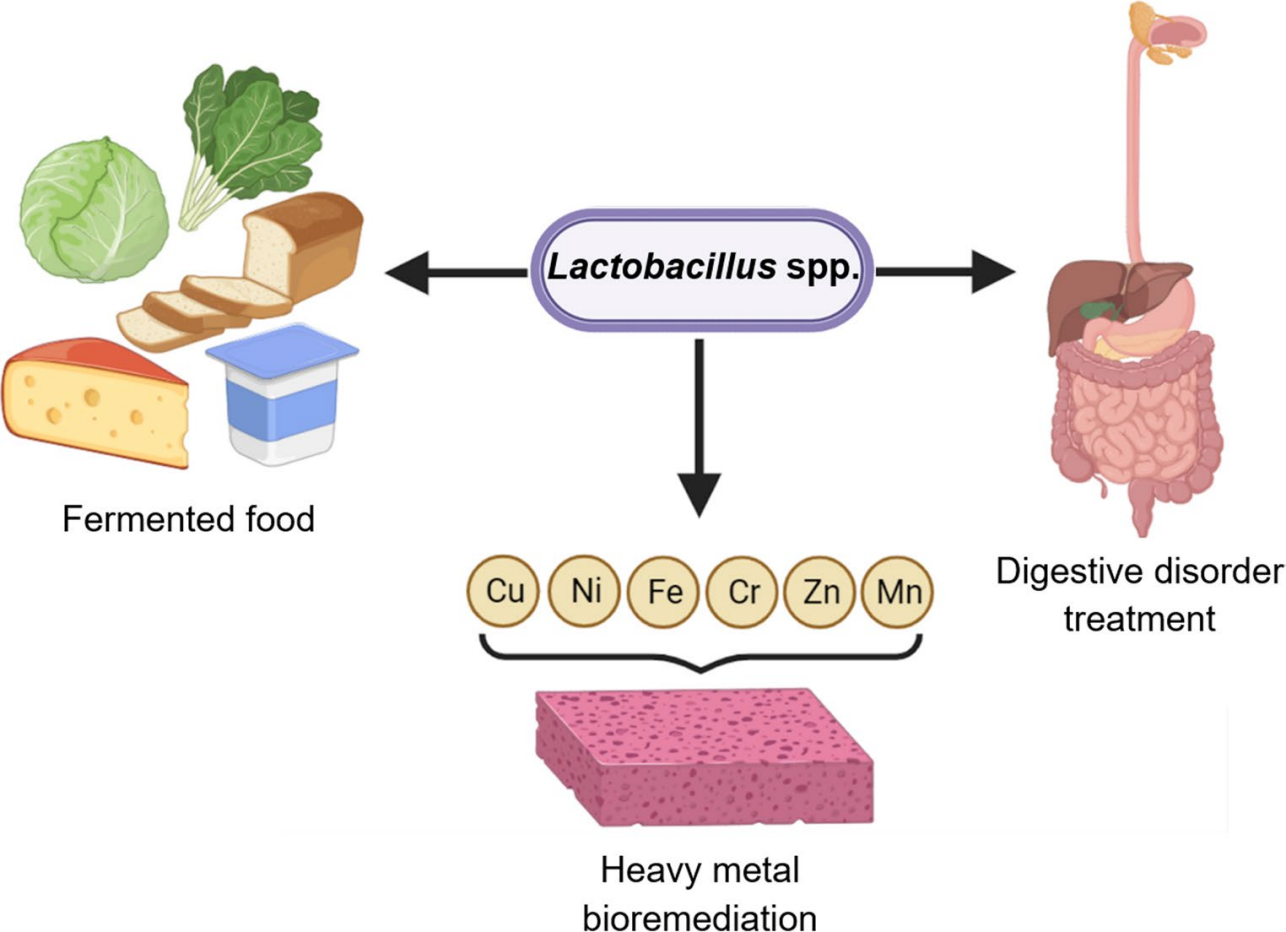
Current applications of *Lactobacillus* spp. (Created with BioRender.com)

The *Lactobacillus* genus, the lactic acid bacteria, comprises gram-positive bacteria characterized by their rod shape, non-spore-forming nature, and facultative anaerobic capacity [32]. *Lactobacillus* constitutes the normal flora in the gastrointestinal tract, oropharynx, and female genitourinary tract [33]. *Lactobacillus* are present in faecal samples either as transiently derived allochthonous strains or native autochthonous strains, accounting for approximately 0.01 to 0.06% of all faecal bacterial species in adults [27, 34, 35]. *Lactobacillus* spp. possess the inherent metabolic capacity to produce lactic acid upon endogenous carbohydrate metabolism and thus, are traditionally used for food fermentation [34]. Probiotic supplements and food products fortified with *Lactobacillus* spp. have demonstrated effectiveness in ameliorating digestive disorders such as lactose intolerance, irritable bowel syndrome, chronic diarrhoea and functional constipation [36–39]. Moreover, *Lactobacillus* have been exploited for their bioremediation potential. *Lactobacillus fermentum* (*L. fermentum*) and *Lactobacillus plantarum* (*L. plantarum*) strains demonstrate resistance to and rapid biosorption against heavy metals such as nickel in various sources, including aquatic waters, industrial wastewater effluents, and soil [40–42]. Figure 1 summarizes the current applications of *Lactobacillus* spp.

Numerous clinical studies have compared the differential gut microbiota profile between T2DM patients and healthy controls from various countries. In a Southern Chinese population, a higher abundance of total *Lactobacillus*, including *Lactobacillus acidophilus* (*L. acidophilus*), *Lactobacillus bulgaricus* (*L. bulgaricus*), and *Lactobacillus rhamnosus* (*L. rhamnosus*), was reported in age- and gender-adjusted T2DM patients [43]. This trend has been consistently observed in multiple populations, in either newly diagnosed or long-standing diabetic patients as summarized in Table 1 [44–51]. A study reported a significantly higher faecal *Lactobacillus* count in diabetic patients, but the significant effect relationship was nullified after further adjustment for fibre intake [48]. A factor for this enrichment is the abnormally high glucose availability that provides a stimulatory environment for the metabolically flexible *Lactobacillus* [46].

**Table 1:** Clinical and observational studies reporting higher Lactobacillus spp. abundance in individuals with type 2 diabetes mellitus (T2DM) compared with non-diabetic controls. The table summarizes the targeted *Lactobacillus* abundance, as indicated by the higher relative abundance in T2DM patients compared to healthy controls based on the sample sizes, study populations, exposure to treatment regimens, main outcomes and sample sources

Target	Sample size	Study population	Recipient of treatment regimens	Relative abundance	Main outcomes	Sample source	Reference
Total *Lactobacillus*	T2DM: 50 Control: 30	Southern China	Only a portion of subjects (not inclusion criteria)	Increased	Higher abundance associated with T2DM	Faecal	[43]
Total *Lactobacillus*	T2DM: 50 Control: 50	Japan	Only a portion of subjects (not inclusion criteria)	Increased	Dysbiosis in T2DM	Faecal	[44]
*Lactobacillus*	T2DM: 40 Control: 13	Italy	Yes	Increased	Altered microbiota in T2DM	Faecal	[45]
*Lactobacillus*	T2DM: 16 Control: 19	India	Yes (known diabetics)	Increased	Correlated with hyperglycaemia	Faecal	[46]
*L. acidophilus* ATCC 4356 (standard strain)	T2DM: 18 Control: 18	Iran	Not stated	Increased	Higher abundance in T2DM	Faecal	[47]
*Lactobacillus gasseri* DSM 20243 T (standard strain	T2DM: 50 Control: 50	Taiwan	No (newly diagnosed diabetics)	Initially increased; lost after fibre adjustment	Diet strongly modified abundance	Faecal	[48]
*Lactobacillus* group	T2DM: 49 Control: 40	Not mentioned	Only a portion of subjects (not inclusion criteria)	Increased	Genus-level expansion in T2DM	Faecal	[49]
*Lactobacillus*	T2DM: 11 Control: 35	India	No (newly diagnosed diabetics)	Increased	Higher abundance in early disease	Faecal	[50]
*Lactobacillus*	T2DM: 87 Control: 49	United States (T2DM); not mentioned for control group	Yes	Increased	Altered metabolic phenotype	Urine	[51]

While the consensus suggests a positive correlation between *Lactobacillus* spp. and T2DM, contradicting findings have also been reported. A recent clinical study suggested that low detection frequencies of *Lactobacillus* spp. in faecal samples were correlated with diabetes development [52]. In a Romanian cohort, *Lactobacillus* species were significantly lower in the T2DM group [53]. Similarly, *L. acidophilus*, *L. salivaricus*, and *L. fermentum* were significantly lower in stool samples of Iranian T2DM patients than in healthy subjects [54]. Significantly lower stool *L. acidophilus* count was also detected in T2DM patients from Egypt [55]. Another Romanian study demonstrated a lower abundance of *L. acidophilus*, *L. plantarum*, and *Lactobacillus reuteri* (*L. reuteri*) in T2DM subjects [56]. Yet, the same paper determined no significant changes between the diabetic and control groups in four other *Lactobacillus* subgroups: *L. fermentum*,* Lactobacillus salivaricus* (*L. salivaricus*), *Lactobacillus johnsonii* (*L. johnsonii*), *and Lactobacillus ruminis* (*L. ruminis*) [56].

The gut microbiota encompasses a diverse community of microorganisms, typically the members of the genus *Lactobacillus*, which are the most important probiotic bacteria that live in symbiosis with their host [27]. The interactions between *Lactobacillus* spp. and their hosts are greatly influenced by diet, genetics and lifestyle. This symbiotic relationship is crucial to maintain the development of the metabolic system [57], which contributes to the unique microbiome in each person’s gut. Sedighi et al. (2017) revealed higher levels of *Lactobacillus* were found in T2DM patients as compared to the healthy group. This observation agreed with numerous studies found in Table 1. These findings may be valuable for devising strategies to control T2DM by modifying the gut microbiota, suggesting the potential of leveraging precision medicine based on targeting *Lactobacillus* spp. in the treatment of diabetes.

Together, these observations support the concept of precision microbiome modulation, whereby *Lactobacillus*-based interventions are tailored to an individual’s baseline gut microbiota, metabolic phenotype, dietary intake and medication exposure instead of following uniform response pattern. The heterogeneous patterns of Lactobacillus abundance observed across populations (Table 1) suggest that probiotic efficacy is context-dependent and may reflect adaptive microbial responses to host glucose availability, fibre intake as well as pharmacological treatments.

### Heterogeneity and Confounding Factors in *Lactobacillus* Abundance

The inconsistent associations between *Lactobacillus* and T2DM across studies are likely driven by methodological and biological heterogeneity. Several other factors may contribute to the discrepancy in these findings, namely the small sample size, different inclusion criteria of the study and heterogeneity of the study population. In addition, the use of 16 S rRNA sequencing for the detection of a reference strain may not fully represent the vast microbiome composition and abundance of the genus. Differences between metataxonomics (16 S rRNA gene sequencing) and metagenomics (whole shotgun metagenomic sequencing) may produce different levels of taxonomic resolution and different functional interpretation of microbiome data [58]. As a result, the illustrated trends can be underestimated or overestimated since the host metabolism may be related to a dysbiotic shift in gut microbiota instead of alterations of a single species [48].

Besides that, clinical cohorts often exhibit significant heterogeneity in diet, fibre intake as well as the use of antidiabetic medication such as metformin, which are known to independently modulate gut microbiota composition and function [59, 60]. This complexity is compounded by the substantial strain-level functional diversity within the *Lactobacillus* spp. Consequently, the species-level abundance does not reliably reflect shared metabolic or immunomodulatory traits. Notably, two strains within the same species may differ markedly in their in short-chain fatty acids (SCFA) production profiles and immune effects [61]. Furthermore, geographical location differences in lifestyle, early-life microbial exposures and endemic pathogen burden contribute to inter-population heterogeneity, thereby complicating the generalization of findings [62, 63]. Hence, addressing these limitations requires standardization of the subject selection criteria, recruitment of participants from diverse geographic regions and application high-resolution shotgun sequencing to improve metagenomics analyses. Moreover, prospective longitudinal studies may be more appropriate to elucidate the causal relationship between microbiome alterations and disease development.

Therefore, it is vital to investigate the potential factors which causes the diversity and development of the *Lactobacillus* spp [64]. One of the main factors is the dietary interventions, as human not only provide habitat for their living but also provide them with food. The ingested food which is utilized by *Lactobacillus* spp. may contribute to their growth, and thereby optimizing their impact on metabolic health, or vice versa. It is of evident in a recent systematic review and meta-analysis of randomized controlled trials on symbiotic supplementation, revealing its effects on glucose metabolism among diabetes individual [65]. This study reported an improvement in fasting plasma glucose, insulin concentrations and homeostasis model assessment of insulin resistance (HOMA-IR) in diabetes patients using the symbiotic supplementation, which is a mixture of gut bacteria (probiotics) and substances which can only be metabolised by gut microbiome (prebiotics).

Nonetheless, all these clinical studies explicitly target microbiome to obtain clinical results and improve clinical efficacy. Given that clinical samples were mainly from faecal, this can be utilised as diagnostic or prognostic tools [66]. Hence, assessment of the gut microbiome’s composition, quantity, and the relative abundance of its metabolites could serve as a distinctive predictive marker for different individuals linking to health conditions and behavioural patterns [67–69]. These emergent findings suggest that the analysis of microbiome characteristics may offer valuable insights into personalized medicine and contribute to more effective diagnostics and prognostics for individual with diabetes.

## Antidiabetic Properties of *Lactobacillus* Spp

### Role of *Lactobacillus* spp. in Modulating Glucose Metabolism and Transport

*Lactobacillus* isolates can exert therapeutic effects against T2DM by regulating the expression and activity of glucose metabolism enzymes and transporters. Upon food intake, starches are hydrolyzed to disaccharides and oligosaccharides by α-amylase before subsequent conversion by α-glucosidase into monosaccharides [70]. Therefore, inhibiting these enzymes limits carbohydrate absorption to enable better glycaemic control, which would be reflected by a proportional decrease in blood glucose levels. From recent findings, *L. plantarum* strains MG4229, MG4296, and MG5025, *Lacticaseibacillus paracasei* (*L. paracasei*) MG5012, *Lactobacillus sakei* Probio65, and *L. plantarum* Probio-093 exhibited potent inhibition to the enzymatic activities of α-glucosidase and α-amylase, which were on par with acarbose [70, 71].

The glucagon signalling pathway is also crucial for the regulation of glucose metabolism. Glucagon binds and activates glucagon receptor (GCGR), which activates the downstream mediators heterotrimeric Gs protein alpha-subunit (Gnas), cAMP-dependent protein kinase (PKA), and CREB-regulated transcriptional coactivator 2 (CRTC2) [72]. CRTC2 subsequently promotes the transcriptional activity of CREB to upregulate the expression of phosphoenolpyruvate carboxykinase (PEPCK) and glucose-6-phosphatase (G6Pase), the rate-limiting enzymes of hepatic gluconeogenesis [73]. T2DM mice treated with *L. rhamnosus* LRa05 displayed diminished expression of several key mediators of the hepatic glucagon signalling pathway, including glucagon, GCGR, Gnas, PKA, CRTC2, PEPCK, and G6Pase [74].

Similarly, *Lactobacillus casei* (*L. casei*) LC89 downregulated the expression of GCGR, Gnas, PKA, CRTC2, PEPCK, and G6Pase in diabetic rats [75]. *L. plantarum* HAC01 supplementation to T2DM mice downregulated PEPCK and G6Pase [76]. Inhibition of the glucagon signalling pathway and gluconeogenesis decrease endogenous glucose production and reduce FBG [75]. *L. acidophilus* KLDS1.0901 and KLDS1.1003 promoted hepatic glycogen synthesis by downregulating glycogen synthase kinase-3 beta (GSK-3β) expression, which also lowered the FBG in T2DM mice [77].

*L. plantarum* PCS26 significantly lowered glucose transporter 2 (GLUT-2) protein expression in Caco-2 cells, decreasing the rate of transepithelial glucose transport [78]. *L. reuteri* GL-104 and *L. salivarius* AP-32 also inhibited hexose uptake and demonstrated significant hypoglycaemic effects in Caco-2 cells by promoting high monosaccharide consumption efficiency and lowering the expression of the intestinal hexose transporter SGLT1 [79]. Similarly, *L. plantarum* inhibited SGLT2 expression and was associated with improved renal insulin sensitivity [80].

Overall, these studies show that selected *Lactobacillus* strains may improve glycaemic control through several mechanism. They may reduce carbohydrate breakdown, lower hepatic gluconeogenesis and limit glucose transportation in the gut. Together, these effects influence glucose absorption and endogenous glucose production, which indicate that probiotics act at several metabolic checkpoints to regulate blood glucose levels.

### Role of *Lactobacillus* spp. in Modulating Inflammation

Hyperglycaemia is known to invoke an inflammatory response by stimulating the secretion of corresponding cytokines [81]. Likewise, a study has suggested the role of inflammatory mediators in insulin resistance development and T2DM pathogenesis [82]. A study in the Kashmiri population reported higher serum levels of tumour necrosis factor-alpha (TNF-α), C-reactive protein (CRP), and interleukin-6 (IL-6) but lower IL-10 concentrations in T2DM patients [82]. The elevated CRP, TNF-α, and IL-6 levels were strongly associated with insulin resistance in diabetic patients from Faisalabad [83]. A study in a Korean cohort reported similar findings, where CRP was significantly associated with incident T2DM irrespective of gender [84]. Meanwhile, CRP is likely a hyperglycaemic by product rather than an etiological biomarker of T2DM [85].

The inflammatory cytokines TNF-α, IL-1β, and IL-6 play significant roles in impairing insulin signalling, contributing to the development and progression of T2DM. A study has shown that TNF-α phosphorylates and inactivates the serine residue of insulin receptor substrate 1, reducing GLUT-4 translocation and impairing the insulin signalling pathway [86]. TNF-α and IL-1β induce the expression of IL-6, which, at high concentrations, stimulates the expression and activity of insulin-degrading enzyme to promote insulin clearance [87–89]. Additionally, TNF-α and IL-1β induce inflammation in peripheral tissues, resulting in the chemotactic invasion and infiltration of immune cells that compromise the structural integrity of these target tissues [90]. Overall, addressing the inflammatory response may ameliorate insulin resistance and hinder T2DM progression.

Herein, the anti-inflammatory potential of *Lactobacillus* spp. in ameliorating the pathological state of T2DM was observed in different *in vivo* models. *L. casei* CCFM419 significantly decreased serum levels of TNF-α and IL-6 but not IL-10 in high-fat and streptozotocin-induced C57BL/6J mice [91]. *L. casei* CCFM419 increased acetic acid and total SCFA, especially butyrate [91]. SCFAs have been postulated to actuate free fatty acid receptors 2 and 3 to initiate inhibitory signalling cascades against inflammation and inhibit histone deacetylase to reduce transcription of pro-inflammatory genes [92]. Meanwhile, *Lactobacillus gasseri* (*L. gasseri*) CKCC1913 reduced the expression of IL-6 and TNF-α in high-fat diet-induced insulin-resistant C57BL/6J mice [93]. In a T2DM zebrafish model, *L. rhamnosus* GG supplementation downregulated the relative expressions of IL-1β and TNF-α, which correlated positively to blood glucose levels [94].

In streptozotocin (STZ)-induced diabetic rats, *L. rhamnosus* Hao9 and *L. fermentum* MCC2759 and MCC2760 lowered the serum concentration and gene expression levels of IL-1β, IL-6, and TNF-α and stimulated IL-10 expression [95, 96]. *L. fermentum* MCC2759 and MCC2760 also reversed hepatic inflammatory infiltration and steatosis formation and normalized glomerular and intestinal integrity [95]. The immunomodulatory function of these strains was postulated to be mediated by modulating the gut microbiota, which is associated with reduced lipopolysaccharide production and improved gut barrier function [95]. It is suggested that IL-10 suppresses inflammatory cytokine generation and improves insulin signalling and glucose metabolism in skeletal muscle [97].

Meanwhile, *L. rhamnosus* Hao9 restores intestinal barrier integrity, preventing the vascular leakage of lipopolysaccharides and pro-inflammatory cytokines [96]. Supplementing high fructose-fed rats with *L. plantarum* significantly decreased renal tissue levels of IL-1β, IL-6, IL-10, and TNF-α but not nuclear factor kappa B (NF-κB) [80]. On the other hand, the anti-inflammatory effect of *Lactobacillus helveticus* (*L. helveticus*) was limited to the decrease in renal TNF-α level [80].

Another study has shown that the heat-killed *L. plantarum* L-137 attenuated systemic inflammation by lowering cytokine expression and improved insulin signalling by increasing protein kinase B (Akt) phosphorylation in the adipose tissue of obese rats [98]. Besides this, *L. plantarum* LRCC5310 and LRCC5314 alleviated insulin resistance in T2DM mice under chronic cold stress by markedly lowering TNF-α, IL-6, and C-C motif chemokine ligand 2 (CCL2) expression, thereby suppressing the pro-inflammatory activity [99]. Meanwhile, *L. paracasei* HII01 significantly increased Akt phosphorylation and GLUT-4 expression and improved insulin signalling by reducing systemic inflammation biomarkers TNF-α and NF-kB in diabetic rats [100]. *L. gasseri* CKCC1913 increased glucose tolerance and reduced the expression of TNF-α and IL-6 [93].

On the other hands, *L. acidophilus* KLDS1.1003 and KLDS1.0901 ablated the levels of pro-inflammation cytokines TNF-α, IL-8, and IL-1β in the liver and colon of T2DM mice [77]. Another study demonstrated that *L. fermentum* MCC3216 significantly lowered IL-6 and elevated IL-10 levels in high fructose-fed T2DM mice [101]. Similarly, *L. fermentum* TKSN041 increased IL-10 and decreased IL-1β and IL-6 in STZ-induced T2DM rats [102]. The strain also upregulated NFκB-inhibitor-α (IκB-α) expression to prevent the activation of NFκB-p65, resulting in lower TNF-α levels [102]. *L. casei* CCFM0412 protected against the pathological inflammatory state induced in T2DM mice, as exhibited by the lowered TNF-α and higher IL-10 levels [103].

Hence, based on these findings as shown in Table 2, *Lactobacillus* spp. may have the potential to improve the pathological state of T2DM by modulating host’s immune response via regulation of pro- and anti-inflammatory cytokine production. Altering the balance of these anti-inflammatory profiles may help to enhance insulin signalling pathway and glucose metabolism, preserving β-cell integrity as well as ameliorate insulin resistance to better control of glucose levels. This immunomodulatory mechanism highlights the potential of strain-specific *Lactobacillus* formulations as adjunctive therapies in the personalized management of T2DM, which reduce chronic low-grade inflammation associated with metabolic dysfunction. However, these findings are based on small-scale animal experiments which require further research involving clinical studies in humans to confirm their relevance and effectiveness in human populations.

**Table 2:** Summary of experimental studies investigate the role of *Lactobacillus* spp. In modulating inflammation in metabolic disease models relevant to T2DM. The table highlights strain-specific effects on inflammatory cytokines, signaling pathways and metabolic outcomes across *in vivo* and *in vitro* models

Strain	Model	Dose, duration and route of administration	Cytokines Affected	Key Findings	References
*L. casei* CCFM419	HFD + STZ-induced mice	1 × 10⁹CFU/mL; 12 weeks; intragastric	↓ TNF-α, ↓ IL-6, ↔ IL-10	Increased SCFAs (esp. butyrate); anti-inflammatory effect via SCFA pathways	[91]
*L. gasseri* CKCC1913	HFD-induced mice	1 × 10⁹ CFU/mL; 6 weeks; oral gavage	↓ TNF-α, ↓ IL-6	Improved glucose tolerance and reduced inflammation	[93]
*L. rhamnosus* GG	T2DM zebrafish	Dose not reported; 10 days; oral administration	↓ TNF-α, ↓ IL-1β	Cytokine reduction correlated with lower blood glucose	[94]
*L. fermentum* MCC2759/2760	STZ-induced rats	10⁹ CFU/mL; 4 weeks; intragastric	↓ TNF-α, ↓ IL-6, ↓ IL-1β, ↑ IL-10	Reversed hepatic inflammation, improved gut barrier integrity	[95]
*L. plantarum*, *L. helveticus*	High fructose-fed rats	10⁹ CFU per 100 g body weight; 6 weeks; gastric gavage	↓ IL-1β, ↓ IL-6, ↓ IL-10, ↓ TNF-α (limited for *L. helveticus*)	Anti-inflammatory effects; *L. helveticus* effect limited to TNF-α	[80]
*Heat-killed L. plantarum* L-137	Obese rats	2 mg/kg/day (low dose) or 75 mg/kg/day (high dose); 4 weeks; oral gavage	↓ Systemic cytokine expression	Improved insulin signalling via ↑ Akt phosphorylation	[98]
*L. plantarum* LRCC5310/5314	Cold-stressed T2DM mice	L. plantarum LRCC5310: 1 × 10⁸ CFU or 1 × 10¹⁰ CFU L. plantarum LRCC5314: 1 × 10⁸ CFU or 1 × 10¹⁰ CFU; 12 weeks; oral administration	↓ TNF-α, ↓ IL-6, ↓ CCL2	Relieved insulin resistance by suppressing inflammation	[99]
*L. paracasei* HII01	Diabetic rats	1 × 10^8^ CFU/day; 12 weeks; oral administration	↓ TNF-α, ↓ NF-κB	↑ Akt phosphorylation and GLUT-4 expression, improved insulin signalling	[100]
*L. acidophilus* KLDS1.1003/0901	T2DM mice	1 × 10⁹ CFU/day; 6 weeks; oral gavage	↓ TNF-α, ↓ IL-1β, ↓ IL-8	Reduced inflammation in liver and colon	[77]
*L. fermentum* MCC3216	High fructose-fed mice	2.0 × 10⁸ CFU/mL; 8 weeks; oral	↓ IL-6, ↑ IL-10	Balanced inflammatory response	[101]
*L. fermentum* TKSN041	STZ-induced rats	1.0 × 10⁹ CFU/mL; 13 weeks; oral gavage	↓ IL-1β, ↓ IL-6, ↑ IL-10, ↓ TNF-α	Upregulated IκB-α to block NF-κB activation	[102]
*L. casei* CCFM0412	T2DM mice	1 × 10⁹ CFU/day; 12 weeks; oral administration	↓ TNF-α, ↑ IL-10	Protected against pathological inflammation	[103]

Collectively, the studies summarized in Table 2 suggest that selected *Lactobacillus* strains can attenuate T2DM-related inflammation by shifting the balance away from pro-inflammatory mediators. This is crucial to glycaemic control because chronic low-grade inflammation disrupts insulin signalling and contributes to systemic insulin resistance. This immunomodulatory mechanism highlights the potential of strain-specific *Lactobacillus* formulations as adjunctive therapies in the personalized management of T2DM, highlighting the importance of precision microbiome modulation rather than generalized probiotic use.

### Role of *Lactobacillus* spp. in Regulating Oxidative Stress

In T2DM, abnormal hyperglycaemia leads to reactive oxygen species (ROS) overproduction, causing oxidative stress via multiple signalling pathway, including the advanced glycation end products pathway, hexosamine pathway, polyol pathway, and protein kinase C pathway [104]. Increased oxidative stress contributes to impaired glucose metabolism, potentially contributing to the development of T2DM, due to the imbalance of ROS generation and scavenging [104, 105].

Notably, the T2DM patients show higher malondialdehyde (MDA) levels, a marker of lipid damage and superoxide dismutase (SOD) activity, coupled with diminished glutathione (GSH) content and other antioxidant enzyme activity [106, 107]. The elevated total SOD activity alone as an adaptive response does not sufficiently protect against the substantial increase in oxidative stress-induced ROS generation [106]. Pancreatic beta cells are particularly susceptible to oxidative damage due to their limited antioxidant capacity and low antioxidant enzyme content, which can lead to beta cell failure [108]. Furthermore, ROS also inhibits GLUT-4 translocation and hinders insulin-stimulated glucose uptake by activating c-Jun N-terminal kinases (JNKs), suppressing the phosphatidylinositol 3-kinase (PI3K)/Akt signalling pathway, or oxidizing proteins in the insulin-signalling pathway [109–111].

Numerous studies indicate that administering *Lactobacillus* spp. exerts antioxidant effects in animal models, thereby delaying diabetes progression. Recent studies showed that *L. paracasei* L14 and NL41 substantially increased the levels of ROS-scavenging enzymes catalase, glutathione peroxidase (GPX), and SOD as well as reduced MDA concentration, restoring most parameters to baseline levels [112, 113]. On the other hand, *L. paracasei* isolated from Malaysian water kefir grains suppressed the STZ-induced increase in MDA levels and improved glucose tolerance in T2DM mice [114].

Compared to other isolates, *Lactobacillus* isolates from these grains possessed higher levels of antioxidant indicators, including high free radical scavenging activity [115]. The antioxidant strength is primarily linked to the high total phenolic and flavonoid content [115]. *Lactobacillus* spp. can increase the bioavailability of phenolic and flavonoid molecules by acting as hydrogen donors to deactivate free radicals [116]. Another finding reported that *L. casei* CCFM0412 promoted hepatic GSH and SOD levels while reducing MDA and ROS [103]. GSH is a nonenzymatic antioxidant, acting directly to quench free radicals or indirectly as the reducing agent for GPX-catalyzed reactions [117].

*L. rhamnosus* Hao9 promoted the hepatic antioxidant capacity, exemplified by the elevated levels of catalase and SOD in streptozotocin-high fat diet-induced T2DM mice [96]. Meanwhile, *L. rhamnosus* LRa05 exerted comparable effects to metformin in increasing catalase and GSH levels while reducing MDA levels in the liver of T2DM mice [74]. However, unlike *L. rhamnosus* Hao9, *L. rhamnosus* LRa05 did not significantly increase hepatic SOD levels, which may be attributed to the differential antioxidant mechanisms between species [74]. Moreover, supplementation with *L. fermentum* MCC3216 exerted a more substantial reduction in lipid peroxidation and an increase in SOD and catalase protein levels, with comparable effects on GSH concentration in the pancreas of high fructose-fed diabetic Wistar rats as compared to the metformin-treated control group [101].

T2DM rats administered with *L. plantarum* SS18*-*5 increased SOD levels and activity and lowered MDA levels [118]. *L. gasseri* CKCC1913 supplementation yielded similar effects in T2DM mice [93]. *L. plantarum* SCS3 effectively recovered SOD, catalase, and GSH levels, providing antioxidant protection [119]. Similarly, the inactivated cellular content of *L. plantarum* SCS4 lowered ROS and MDA content and restored SOD, catalase, GPX, and GSH concentration to physiological levels [120]. In the kidneys of fructose-fed rats, *L. plantarum* and *L. helveticus* elevated SOD2 expression [121]. Supplementation of these *Lactobacillus* isolates improved glycaemic indices and enhanced insulin sensitivity in the diabetic models [93, 118–120].

Several studies suggested that *Lactobacillus* spp. exert their redox role through multiple mechanisms such as inherent generation of oxidative resistance products, regulation of nuclear factor erythroid 2 (NFE2)-related factor 2 (Nrf-2) signalling, modulation of NF-κB transcription factor activity, and uncoupling of endothelial nitric oxide synthase [122–125]. Numerous investigations revealed that the antioxidant properties of *Lactobacillus* protect peripheral organs from oxidative damage. For instance, *L. paracasei* L14 markedly increased beta-cell number, sustained the structural arrangement of pancreatic islets, and improved the hepatic histological morphology [112]. *L. rhamnosus* Hao9 and *L. rhamnosus* LRa05 improved hepatic injury by reducing oxidative stress-associated fibrosis and lowering vacuolar degeneration, respectively [74, 96]. Despite these promising results in the animal models, further clinical investigations are necessary to verify the therapeutic efficacy in humans.

As summarized in Table 3, these *in vivo* findings demonstrate that *Lactobacillus* spp. supplementation ameliorates oxidative stress-induced insulin resistance and hyperglycaemia by upregulating ROS-scavenging enzymes or nonenzymatic antioxidants as well as diminishing ROS production. These results also highlight the antioxidant defences such as SOD, catalase, and GSH. Hence, it shows that oxidative stress as a modifiable target via microbiome-based approaches. Nevertheless, the antioxidant effects differ across strains whereby strain selection remains essential for a precision microbiome modulation.

**Table 3:** Summary of experimental studies evaluating the effects of *Lactobacillus* spp. on oxidative stress regulation in animal models of T2DM and metabolic syndrome. The table outlines strain-specific influences on enzymatic and non-enzymatic antioxidant systems and reactive oxygen species (ROS) generation

*Lactobacillus *species	Animal model (disease state)	Dose, duration and route of administration	Effect on enzymatic antioxidant	Effect on non-enzymatic antioxidant	Effect on ROS generation	Reference
*L. casei *CCFM0412	C57BL/6 J mice (HFD/STZ-induced T2DM)	1 × 10⁹ CFU/day; 12 weeks; oral administration	Increase SOD	Increase GSH	Reduce MDA and ROS	[10]
*L. helveticus*	Wistar rats (high fructose-induced metabolic syndrome)	1 × 10⁹ CFU/100 g body weight; 6 weeks; gastric gavage	Increase SOD2	-	-	[121]
*L. fermentum* MCC3216	Wistar rats (high fructose-induced T2DM)	2.0 × 10⁸ CFU/mL; 8 weeks; oral	Increase SOD and catalase	Increase GSH	Reduce lipid peroxidation	[101]
*L. paracasei *NL41	Sprague–Dawley rats (HFD/STZ-induced T2DM)	1 × 10¹⁰ CFU/day; 12 weeks; oral gavage	Increase catalase and SOD content	Increase GPX	Reduce MDA concentration	[113]
*L. rhamnosus *LRa05	Kunming mice (HFD/STZ-induced T2DM)	1 × 10⁹ CFU/day; 6 weeks; oral gavage	Increase SOD (not significant), decrease catalase	Decrease GSH	Reduce MDA	[74]
*L. plantarum *SS18*-*5	Sprague Dawley rats (HGFD/STZ-induced T2DM)	1 × 10¹⁰ CFU/day; 6 weeks; oral gavage	Increase SOD	-	Reduce MDA concentration	[118]
*L. plantarum* SCS4 (inactivated cellular content)	Kunming mice (STZ-induced T2DM)	2 × 10⁹ CFU/dose; 10 weeks; oral gavage	Increase SOD and catalase	Increase GPX and GSH	Reduce ROS and MDA concentration	[120]
*L. plantarum* SCS3	Kunming mice (STZ-induced T2DM)	5 × 10⁹ CFU/day; 10 weeks; intragastric administration	Increase SOD and catalase	Increase GSH	-	[119]
*L. rhamnosus *Hao9	C57BL/6J mice (HFD/STZ-induced T2DM)	1 × 10⁹ CFU/day; 11 weeks; oral gavage	Increase catalase and SOD	-	-	[96]
*L. gasseri* CKCC1913	C57BL/6J mice (HFD/STZ-induced T2DM)	1 × 10⁹ CFU/mL; 6 weeks; oral gavage	Increase SOD	-	Reduce MDA concentration	[93]
*L. paracasei *(Malaysian water kefir grains)	C57BL/6 mice (HFD/STZ-induced T2DM)	Low dose (LD): 1 × 10⁶ CFU/mL; High dose (HD): 1 × 10¹⁰ CFU/mL; 4 weeks; oral gavage	-	-	Reduce MDA concentration	[114]
*L. paracasei *L14	Sprague–Dawley rats (HFD/STZ-induced T2DM)	1 × 10¹⁰ CFU/day; 12 weeks; oral gavage	Increase catalase and SOD content	Increase GPX	Reduce MDA concentration	[112]

*Abbreviations:HFD* high-fat diet, *STZ* streptozotocin, *SOD* superoxide dismutase, *GSH* glutathione, *MDA* malondialdehyde, *ROS* reactive oxygen species, *GPX* glutathione peroxidase, *HGFD* high glucose fat diet

### Role of Lactobacillus spp. in Remodelling Diabetic Gut Microbiota

*Lactobacillus* spp. augments microbial diversity and richness, as indicated by the increase in Shannon index and Chao1 scores [74, 77, 126]. At the phylum level, the Firmicutes/Bacteroidetes (F/B) ratio is significantly lower upon the administration of *L. acidophilus* KLDS1.0901 and KLDS1.1003, *L. casei* CCFM419, *L. casei* LC89, *L. plantarum* LRCC5314, *L. plantarum* SHY130, *L. rhamnose us* LRa05, and *L. paracasei* IMC 502 in T2DM rodent models [74, 75, 77, 91, 126–128]. In contrast, the F/B ratio of T2DM patients typically exhibit a higher F/B ratio, with Firmicutes associated with insulin resistance and Bacteroidetes with improved glucose tolerance [50, 129–131]. While the exact mechanism has not been elucidated, the altered F/B ratio may lead to T2DM development by modulating inflammation.

Interestingly, Firmicutes perform saccharolytic fermentation, which may increase the gastroepithelial barrier permeability and induce inflammation, as indicated by its positive correlation with inflammatory markers TNF-α and IL-1β in obese and T2DM patients, respectively [132–134]. However, the Firmicutes phylum also comprises most of the butyrate-producers [135]. This paradox could be due to a reduction in butyrate-producing bacteria, which are replaced by other Firmicutes, leading to lower levels of the anti-inflammatory SCFA [136]. Therefore, restoring the F/B ratio and composition may normalize the SCFA levels and alleviate inflammation and thus improve insulin sensitivity.

Increase abundance of *Parasutterella* has been observed in the gut microbiota of T2DM patients [137, 138]. The administration of *L. paracasei* strains L14 and NL41 to T2DM rodent models reduced *Parasutterella* levels [112, 139]. Various studies have linked higher blood glucose levels and improved insulin sensitivity with increased Parasutterella in diabetic animals, supporting its pro-diabetic role [140–142]. As a high L-cysteine consumer, the high *Parasutterella* abundance reduces serum levels of this metabolite [137]. The inverse relationship between *Parasutterella* and L-cysteine levels may contribute to the pathogenesis of T2DM. L-cysteine supplementation improves glucose utilization by promoting the secretion of insulin-sensitizer adiponectin and reduces oxidative stress by increasing GSH levels and lowering ROS [143]. Therefore, by reducing *Parasutterella* abundance, the *L. paracasei* strains increase serum L-cysteine levels, which decreases oxidative damage to pancreatic beta cells and alleviates insulin resistance [137].

**Fig. 2:**
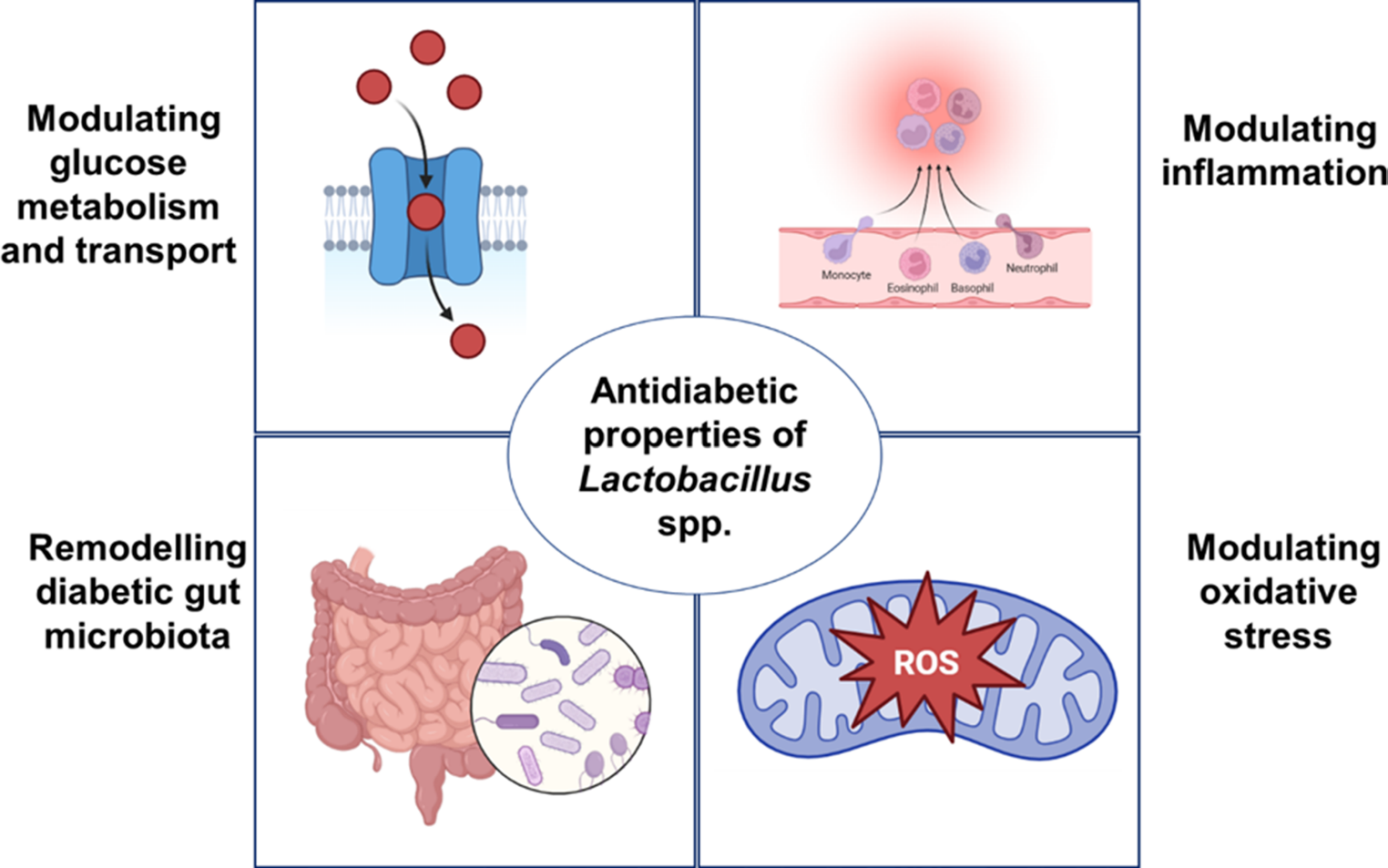
Antidiabetic properties of lactobacillus spp. which play important roles in managing diabetes by enhancing glucose uptake, reducing inflammation, supporting gut microbiota balance and lowering oxidative stress. (Created with BioRender.com)

Lactobacillus species play important roles in managing diabetes by enhancing glucose uptake, reducing inflammation, lowering oxidative stress as well as supporting gut microbiota balance as shown in Fig. 2. Specific strains such as L. *paracasei* L14 and NL41 increased the abundance of the *Lachnospiraceae NK4A136* group [112, 139]. *L. rhamnosus* LRa05 increased the abundance of *Akkermansia* [74]. Microbiota analyses of T2DM subjects and animal models have shown reduced abundance in both genera [142, 144, 145]. *Lachnospiraceae NK4A136 group* and *Akkermansia* are negatively associated with fasting blood glucose and HbA1c (glycated haemoglobin), respectively [142, 146]. The established correlation suggests that restoration of the genera abundance may mitigate the pathological state of T2DM.

Intervention with metformin, the gold standard drug for T2DM, also induced similar effects by increasing their abundance in high-fat diet mice, further supporting their beneficial roles [147]. *Lachnospiraceae NK4A136 group* and *Akkermansia* are butyrate-producers [148, 149]. Butyrate promotes intestinal barrier integrity by increasing the expression of claudins, the main constituents of tight junctions [150]. Additionally, as mucin-degrading bacteria, *Akkermansia* increases the number of goblet cells, stimulating mucus synthesis [151]. Hence, an enriched abundance of *Akkermansia* and *Lachnospiraceae NK4A136 group* reduces intestinal permeability, prevents lipopolysaccharide entry to the vascular system, and reduces inflammation, ultimately alleviating glucose intolerance.

Table 4 summarizes the microbiota changes induced by various *Lactobacillus* spp. and the corresponding roles. Many studies reported that increased SCFA-producing taxa, improved in gut barrier function and reduced inflammatory leakage. These microbiota shifts are associated with lower inflammation and improve insulin sensitivity. While the consensus agrees that *Lactobacillus* spp. alter the microbiome composition to improve glycaemic control, the magnitude of the microbial community shifting may vary based on the specific strain and dose of probiotic administered. These strain-specific and ecosystem-dependent microbiota shifts supports the importance of precision microbiome modulation, in which targeted *Lactobacillus* interventions are aligned with host microbiome structure and functional capacity.

**Table 4:** Effects of Lactobacillus spp. on gut microbiota composition and functional profiles in animal models.  This table summarizes strain-dependent alterations in key microbial taxa and community structure, as well as the associated metabolic and immunomodulatory roles, demonstrating how *Lactobacillus* contributes to microbiota remodeling as a strategy for precision intervention in T2DM

*Lactobacillus *spp.	Animal model (disease state)	Dose, duration and route of administration	Changes in microbiota	Roles	References
*L. casei* CCFM419	C57BL/6J mice (HFD/STZ-induced T2DM)	1 × 10⁹CFU/mL; 12 weeks; intragastric	Lower Firmicutes/Bacteroidetes (F/B) ratio; increased total SCFAs	Modulates gut flora–SCFA–inflammation/GLP-1 axis, reduces inflammatory cytokines (TNF-α, IL-6) and improves insulin resistance	[91]
*L. acidophilus* KLDS1.1003 and KLDS1.0901	C57BL/6J mice (HFD/STZ-induced T2DM)	1 × 10⁹ CFU/day; 6 weeks; oral gavage	Reduced F/B ratio; enrichment of SCFA-producing taxa	Rebalances gut microbiota, normalizes SCFA levels, improves glucose and lipid metabolism	[77]
*L. rhamnosus* LRa05	Kunming mice (HFD/STZ-induced T2DM)	1 × 10⁹ CFU/day; 6 weeks; oral gavage	Increased SCFA-producing genera (*Alloprevotella*, *Bacteroides*); decreased proinflammatory genera (*Odoribacter*, *Mucispirillum*).	Ameliorates hyperglycaemia and insulin resistance by modulating glucagon-mediated signalling, reducing hepatic oxidative stress and LPS-related inflammation, and partially reshaping gut microbiota toward more SCFA-producing taxa	[74]
*L. casei* LC89	HFD/STZ-induced T2DM mice	1 × 10⁹ CFU/day; 6 weeks; oral gavage	Reduced F/B ratio; modulation of gut community structure towards SCFA-producing genera	Improves hepatic glucagon response and insulin sensitivity via microbiota remodelling	[75]
Heat-killed* L. plantarum LRCC5314 (HK-LRCC5314*	C57BL/6 mice (cold stress and HFD-induced T2DM)	1 × 10⁸ CFU/day; 12 weeks; oral administration	Altered gut community structure; enrichment of beneficial and butyrate-producing genera (*Barnesiella*, *Alistipes*, *Akkermansia*) and reduction of potentially pro-inflammatory genera (*Ruminococcus, Dorea, Clostridium*).	Relieves stress-related hyperglycaemia and insulin resistance and lowers inflammatory markers via gut microbiome modulation	[126]
*L. plantarum* SHY130	C57BL/6J mice (HFD/STZ-induced T2DM)	10¹⁰ CFU/kg body weight per day; 10 weeks; oral gavage	Readjusts intestinal flora structure; enriches SCFA-producing genera (*Faecalibaculum, Odoribacter, Alistipes*); increases colonic SCFA levels.	Supports enteroinsular axis regulation and glycaemic control by increasing SCFA-producing bacteria, upregulating colonic SCFA receptors GPR41/GPR43, and protecting islet α/β-cell balance	[127]
*L. paracasei* IMC 502	C57BL/6J mice (HFD/STZ-induced T2DM)	1 × 10⁹ CFU/day; 15 weeks; oral administration	Reduced F/B ratio; enrichment of SCFA-producing genera	Mediates the gut microbiota–SCFA–hormone/inflammation pathway to ameliorate T2DM	[12]
*L. paracasei* NL41	Sprague–Dawley rats (HFD/STZ-induced T2DM)	1 × 10¹⁰ CFU/day; 12 weeks; oral gavage	Regulates intestinal microbiota and inflammatory response (unpublished data)	Decreases insulin resistance and oxidative stress, protects β-cells, and improves glycaemic and lipid metabolism	[113]
*L. paracasei *L14	*L. paracasei *L14	1 × 10¹⁰ CFU/day; 12 weeks; oral gavage	Sprague–Dawley rats (HFD/STZ-induced T2DM)	Decreased *Parasutterella*; increased *Lachnospiraceae *NK4A136 group	[112]

*Abbreviations:HFD* high-fat diet, *STZ* streptozotocin, *T2DM* type 2 diabetes mellitus, *SCFA* short-chain fatty acid

## Precision Nutrition Development Using *Lactobacillus* spp. for T2DMz

Leveraging advancements in precision medicine, which focuses on personalized healthcare based on individual variability in genes, environment and lifestyle, has driven the recent precision and personalized nutrition strategies [152, 153]. Numerous studies reported that different probiotic strains of *Lactobacillus* may play a pivotal role in modulating host glycaemic responses, oxidative stress pathways and inflammatory states.

### Preclinical Evidence

At the preclinical level, multiple studies demonstrate that fermented foods containing *Lactobacillus* spp. exert antioxidant and glucose-regulating effects in animal models. A study has shown that T2DM mice treated with defatted rice bran fermented by *L. fermentum* MF423 exhibited upregulated levels of SOD, total antioxidant capacity (T-AOC) and GPX and diminution of MDA levels [154]. These effects were non-significant upon treatment with unfermented rice bran, demonstrating that *Lactobacillus* fermentation enhanced the antioxidant capacity to confer hepatic protection and improve glucose and lipid metabolism [154]. Similarly, fermentation of bitter melon juice with *L.* fermentum LLB3 exhibited more potent antioxidant capacity in T2DM rats, with a significant increase in SOD levels compared to the nonfermented juice [155]. Comparable findings were observed in T2DM rats treated with *L. plantarum* NCU116-fermented carrot juice, resulted in a greater increase in CAT, GPX, and SOD activities and T-AOC levels than its non-fermented form [156].

Meanwhile, various *Lactobacillus* spp. were found to exhibit regulatory effects on glucose metabolism pathways. The hypoglycaemic effects of *L. plantarum*-fermented rice germ extracts in C57BL/6KsJ-db/+ mice were attributed to the modulation of hepatic glucose metabolism [10]. The fermented extract upregulated the expression of glucokinase to stimulate glucose storage and utilization and downregulated the expression levels of G6Pase and PEPCK to reduce gluconeogenesis [10]. Based on* in vitro* assays, fermentation with *L. plantarum* FNCC 0027 elevated the inhibitory activities of α-amylase, α-glucosidase, and amyloglucosidase in Jamaican cherry juice, which lowers glucose absorption [157]. Similarly, yoghurt fermented with *L. plantarum* KU985438 and *L. rhamnosus* KU985439 significantly reduced α-amylase concentrations in STZ-induced diabetic rats [158]. Black goji berry juice fermented with *L. rhamnosus* GG exhibited more potent DPP-IV inhibitory activity *in vitro* [159]. DPP-IV inhibition prevents the degradation of GLP-1 and increases its endogenous levels, resulting in elevated insulin secretion and lower glucose production to enhance glycaemic control [159]. However, the therapeutic superiority of fermented matrices over stand-alone probiotic supplementation remains inconsistent. A study showed that *L. casei* Q14 fermented yoghurt improved glucose tolerance and insulin levels, downregulated the expression of gluconeogenesis-associated enzymes, and re-established a protective microbiota [160]. The antidiabetic effects of the fermented yoghurt, while more effective than plain yoghurt, were comparable to that of the probiotic-only group, suggesting the strain itself was the primary driver of bioactivity [160]. *L. gasseri* SBT2055-fermented skim milk powder increased the expression of insulin genes and transcription factor *Pdx1*, which promoted glycogen levels in diabetic Goto-Kakizaki rats [161]. The fermented milk powder also alleviated inflammation by suppressing serum levels of pro-inflammatory cytokines TNF-α, macrophage inflammatory protein-1α (MIP-1α), and IL-18 and pancreatic levels of granulocyte colony-stimulating factor (G-CSF) [161]. Nevertheless, these findings suggest that the food matrix may not always enhance the intrinsic metabolic benefits of the Lactobacillus strain. Furthermore, some studies have not shown clear advantages of fermented products over probiotics alone, raising questions about the specific role of fermentation in mediating these benefits.

However, its effects on the diabetic indices, such as postprandial glucose response and plasma insulin levels, were similar to the unfermented equivalent [161]. In diabetic Wistar rats, *L. plantarum* NCU116-fermented *Momordica charantia* juice ameliorated oxidative stress and induced significant hypoglycaemic, hypoinsulinemic, and hypolipidemic effects as compared to the untreated controls [162]. While the fermented juice exhibited more potent antidiabetic effects than the nonfermented counterpart, the differences were also non-significant [162].

### Clinical Evidence

Clinical evidence also supports the potential role of *Lactobacillus* spp. in metabolic management. For instance, current clinical evidences support the usage of *Lactobacillus* strains in improving glycaemic control. A recent randomized clinical trial reported that probiotic *Lactobacillus paracasei* HII01 improved glycaemic control in T2DM patients throughout the 12 weeks of intervention with no serious adverse effects or symptoms among the participants [1]. This is in accordance with another study by Khalili and colleagues who demonstrated that *Lactobacillus casei* could improve glycemic response in patients with T2DM [29]. Despite the different strains of *Lactobacillus* employed in these studies, the findings revealed the potential of utilising *Lactobacillus* spp as an adjunct treatment for T2DM. Furthermore, the effectiveness of *Lactobacillus* spp. in managing T2DM extends to augmenting the properties of functional food, which is associated with enhanced antioxidant effects and enzymatic expression and activity. However, the relative contribution of fermentation versus strain-specific bioactivity remains unclear.

From a practical consumer health perspective, current findings suggest that selected *Lactobacillus*-containing foods or supplements may be utilised as beneficial adjunct therapies to support glycaemic control and attenuate oxidative stress in T2DM. However, it is worth noting that not all the fermented products or probiotic formulations labelled with *Lactobacillus* necessarily exert clinically relevant antidiabetic effects. Therefore, these products should be considered as adjuncts, not substitutes, for standard dietary and pharmacological T2DM management. Their use is best justified when supported by robust clinical evidence for the specific strain, dose and formulation along with ongoing well-controlled studies which are essential to validate and optimise these recommendations.

Overall, advancing precision nutrition with *Lactobacillus* spp. for T2DM requires further exploration on strain–substrate interactions, effectiveness of fermentation and patient-specific responses. In particular, the fermentation-induced bioactivity-enhancing effect may be associated with discrete strains or food types and vary according to the inoculation concentration, treatment dose and treatment duration. The fermentation protocol should also be optimized for each strain as changes in incubation conditions, including time, pH, temperature, and salt concentration, can alter SCFA production [163, 164]. Moreover, prospective studies should investigate the individual and combined effects of the food and specific *Lactobacillus* strain to accurately determine the beneficial effects of *Lactobacillus* fermentation.

**Fig. 3:**
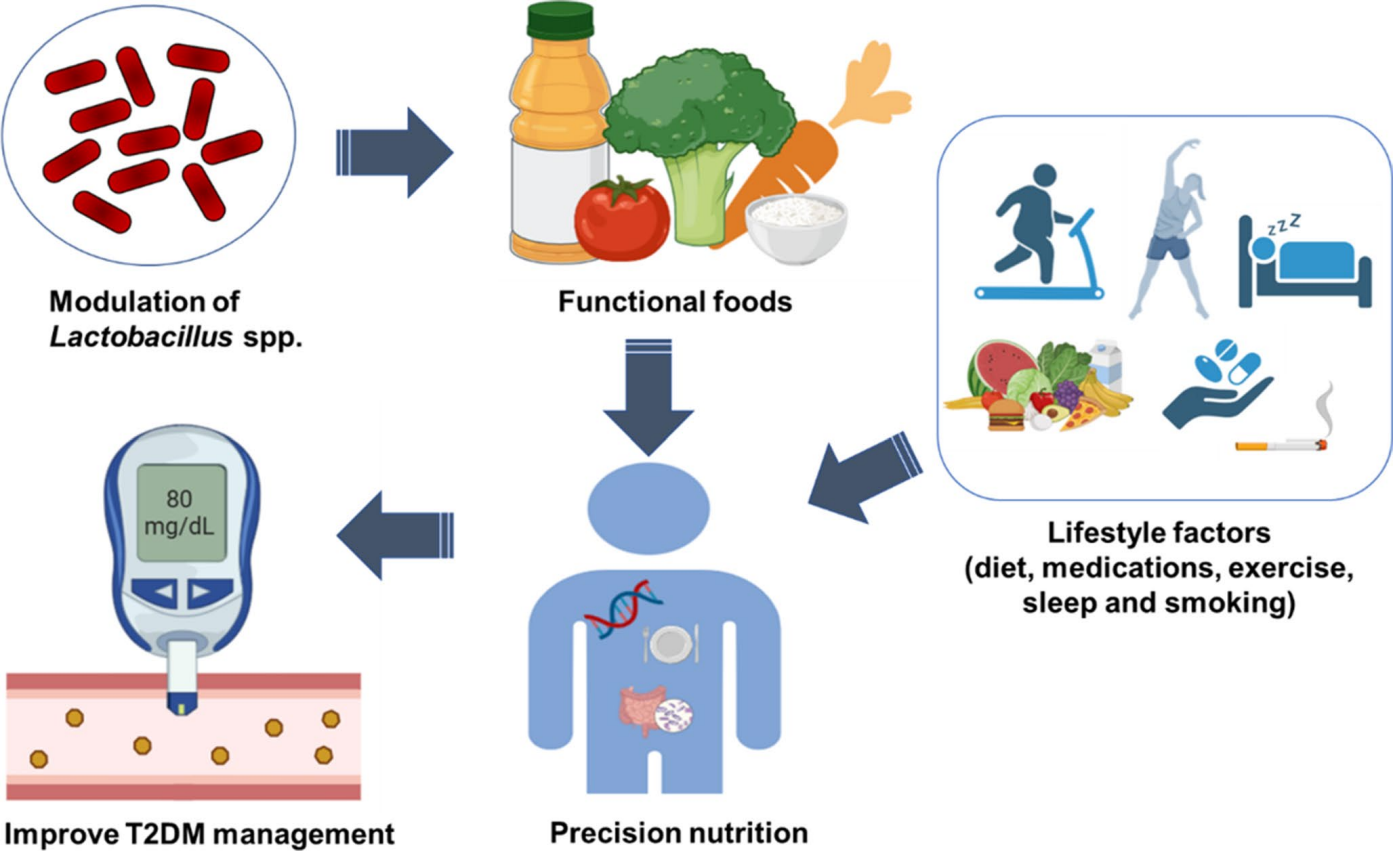
The schematic illustrates how host factors (e.g., genetics and baseline metabolic phenotype) and lifestyle factors (diet, physical activity, smoking, sleep and medication use) interact with gut microbiome features to influence *Lactobacillus* spp. modulation of* Lactobacillus* through targeted functional foods is integrated into a precision-nutrition workflow aimed at optimizing glycemic control and improving T2DM management (Created with BioRender.com)

Additionally, the antidiabetic effects of fermented food products should be explicitly distinguished between their inherent antihyperglycemic properties and the fermentation-induced enhancements by comparing the metabolite content pre- and post-fermentation. Furthermore, to ensure result consistency, a consensus should be drawn to clearly define the primary endpoint as a measure of therapeutic effectiveness. Ultimately, application of strain-specific strategy which integrates both host and microbial factors is crucial to harness the potential of *Lactobacillus*-based precision nutrition in T2DM management as summarised in Fig. 3.

## Challenges and Future Perspectives

### Safety and the Risk of Antimicrobial Resistance

One of the primary challenges in the application of *Lactobacillus*-based probiotics for therapeutic purposes lies in ensuring bacterial viability and stability during gastrointestinal transit. Probiotic treatment involves the consumption of large quantities of bacteria: a minimum dose of 106 CFU bacteria is needed to exert a beneficial effect, but higher administration doses of at least 109 CFU are usually required to account for the ablated bacteria viability due to harsh gastric environments [91]. Several *Lactobacillus* strains resist low pH and bile acids [165, 166]. 

However, not all strains demonstrate such tolerance and exhibit impaired stability and survivability in internal gastric conditions, thus reducing their therapeutic efficacy. Various methods can account for the variability in strain tolerance, including probiotic encapsulation and bioengineering. *Lactobacillus* spp. encapsulation with alginate, pectinate, and spray drying retains high viability [167–170]. A recent study reported that advanced encapsulation approaches using alginate–basil seed mucilage–prebiotic microencapsulation showed markedly improve probiotic *Leuconostoc mesenteroides* ABRIINW.N18 viability in yoghurt during storage and simulated gastrointestinal passage [171]. Hence, these technologies could be adapted for *Lactobacillus* strains used in T2DM interventions to enhance their delivery to distal regions of the gut.

In addition, safety and regulatory challenges pose significant obstacles to the broader clinical implementation of *Lactobacillus* spp. in T2DM management. Although their therapeutic benefits are indisputable, several publications have raised safety concerns about probiotic consumption. Long-term supplementation of a multi-strain *Lactobacillus* mixture in healthy rats induced systemic pro-inflammatory responses, elevated cardiovascular risk, and enlarged colonic lymphoid aggregates [172]. *Lactobacillus* probiotic supplementation has also been associated with sepsis, Lactobacillus endocarditis, and vertebral osteomyelitis [173–175]. 

Due to poor regulation, the vague probiotics formulations marketed are especially detrimental in managing adverse health conditions such as inflammatory bowel diseases or immunosuppressive disorders [176]. Therefore, it is vital to verify the adverse effect profile of various preparations and ensure that the product adheres to the stringent quality requirements and is contamination-free [177]. An innovative method is to extract the intracellular content of *Lactobacillus* probiotics and subsequently inactivate it to prevent septic infection while still retaining efficacy in T2DM amelioration [70].

Nevertheless, navigating the clinical translation of *Lactobacillus* spp. requires heightened regulatory scrutiny following recent updates from the Food and Drug Administration (FDA) and The European Food Safety Authority (EFSA), which increasingly differentiate therapeutic live biotherapeutic products (LBPs) from dietary supplements [178, 179]. Furthermore, a recent large‑scale whole‑genome sequencing analysis of 579 isolates from 12 commonly used probiotic species identified mobile antibiotic‑resistance genes (ARGs) in commercial *Lactobacillus* strains, highlighting potential safety risks and the need for careful safety assessments [180].

Furthermore, probiotics pose a risk as potential reservoirs ARGs. Unlike intrinsic ARGs, the acquired ARGs may be disseminated via horizontal gene transfer in the environment, conferring the resistant trait to potentially pathogenic microbes. Several studies have affirmed the presence of ARG among probiotic *Lactobacillus* strains. The blaTEM resistance gene, encoding extended-spectrum beta-lactamases against β-lactams antibiotics, has been detected in various probiotics-derived *Lactobacillus* isolates [181]. 

Similarly, at least one ARG has been detected in *L*. *plantarum, Lactobacillus* *delbrueckii*, and *L. helveticus* obtained from probiotics and food products [182]. While the ARG detection frequency is relatively lower than other bacterial species, ARGs are potentially transferable since they are plasmid-associated, with those in *L. plantarum* being exceptionally diverse [182]. Therefore, the commercialization of probiotics may invoke selective pressure and favour highly resistant *Lactobacillus* species, contributing to the emergence and spread of antimicrobial resistance.

### Precision Probiotics: Multi-omics Profiling and Personalized Interventions

Despite the promising antidiabetic potential of *Lactobacillus* spp., the strain-specific mechanisms by which *Lactobacillus* spp. modulate glycaemic control remain poorly understood [15]. This impedes the development of precise and targeted probiotic interventions which require further research to comprehend the relationship between *Lactobacillus* spp. and individuals with diabetes. Consequently, it is crucial to identify selection of strains based on their distinct mechanisms of action or biomarkers for response prediction. Thus, integrating comprehensive clinical and multi-omics profiling, including personalized microbiome profiling could identify T2DM subgroups with specific *Lactobacillus* targets and enable personalized probiotic interventions [183, 184]. The integration of diverse high-throughput multi-omics datasets enabling pharmacomicrobiomics encompasses of different modules including amplicon sequencing, metagenomics, metatranscriptomics, metaproteomics and metabolomics/metabonomics [50]. These modules enable researchers to investigate a wide range of gene variation and modulation datasets obtained from multi-omics sequencing. 

With the recent advancement in artificial intelligence, machine learning models and bioinformatics tools can be utlised to individual responses to probiotics and support personalized therapeutic decision-making. These technologies facilitate the development of tailored microbiome interventions by integrating host-specific data such as genetic background, existing gut microbiota composition and metabolic status for the T2DM patients. Moving away from traditional “one-size-fits-all” treatment to precision-based medicine, the current technology leverages the big data from patients’ electronic health records. This includes genomic information, biomarkers, laboratory and radiological investigations data [11, 185, 186]. 

**Fig. 4:**
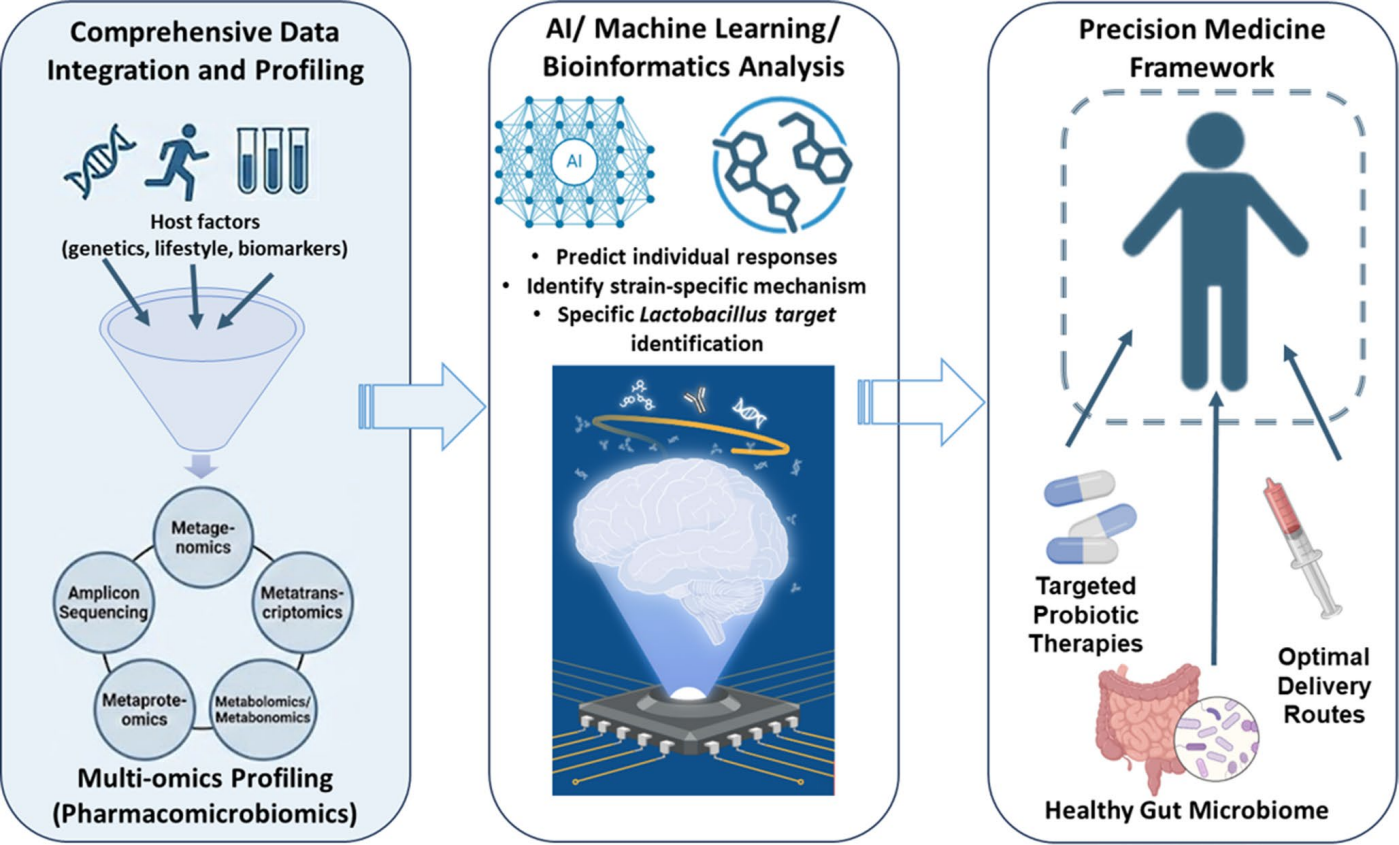
Integrated workflow for developing precision *Lactobacillus* probiotic interventions in T2DM. The process begins with the acquisition of "big data," integrating comprehensive host clinical profiles (genetics, lifestyle) with diverse high-throughput multi-omics datasets. Artificial intelligence (AI), machine learning (ML) and bioinformatics tools process this integrated data to overcome current knowledge gaps regarding strain-specific mechanisms. This analytical step identifies distinct T2DM subgroups and matches them with specific Lactobacillus targets based on predicted response biomarkers. The final outcome is the implementation of tailored microbiome interventions, guiding individualized therapeutic decision-making away from a "one-size-fits-all" model. (Created with BioRender.com)

Hence, precision medicine strategies which assess the patient's genetic profile, lifestyle habits and environmental factors are vital in guiding individualized treatment decisions and predicting therapeutic outcomes. Nevertheless, longitudinal clinical trials are critical to establish causal relationships between specific* Lactobacillus *strains and glycaemic control in T2DM patients [187]. Such trials are essential to translate the promising potential of targeted probiotic therapies into clinical practice by identifying strain-specific indications, optimal dosages, delivery routes as well as safety monitoring under a precision medicine framework. Figure 4 illustrates the shift from traditional probiotic approaches to a precision medicine framework.

### Alternative Microbiome-targeted Strategies: Paraprobiotics, Postbiotics and Fecal Microbiota Transplantation

While the direct administration of live *Lactobacillus* spp. represents a primary strategy for precision microbiome modulation in T2DM management, alternative approaches are rapidly gaining traction to mitigate infection risks while retaining beneficial effects. The current therapeutic landscape is shifting to include non-viable microbial components and metabolites. This has led to the emergence of paraprobiotics (inactivated microbial cells or cell fractions) and postbiotics (bioactive microbial metabolites or cell-free components), representing the next generation of microbiome-based interventions, which offer advantages such as greater stability, safety and a more focused mechanism of action.

Paraprobiotics also referred to as inactivated or heat-killed probiotics, represent non-viable microbial cells or fractions that confer a health benefit to the host [188]. Findings revealed that heat-killed strains of *Lactobacillus* and *Bifidobacterium*, exert their anti-diabetic and metabolic effects primarily through direct immune modulation and gut barrier fortification via their structural component [189]. Various studies showed that paraprobiotics are able to modulate inflammatory and oxidative pathways which helps in improving glucose homeostasis [189]. A current study reported that the purified extracellular polysaccharide (EPS) derived from the novel strain *Lactobacillus acidophilus* YL01 possess significant insulin-sensitizing and anti-obesity properties in high-fat mice via modulating intestinal specific bacterial groups and AMPK/ACC signaling pathway [190]. Overall, non-viable approaches offer therapeutic benefits while reducing the risks associated with live bacteria, providing a more favorable safety profile. 

Meanwhile, postbiotics are defined as “a preparation of inanimate microorganisms and/or their components that confers a health benefit on the host”, according to the International Scientific Association for Probiotics and Prebiotics (ISAPP) in year 2021 [191]. It has shown postbiotics has pharmacological advantages over live probiotics, including superior safety profiles for immunocompromised individuals, enhanced thermal and storage stability as well as precise dosing [192]. A current randomized controlled trial (RCT) has demonstrated that supplementation with a specific *Lactobacillus rhamnosus*-derived postbiotic preparation for three months led to a significant reduction HbA1c and fasting plasma glucose in T2DM patients, primarily attributed to an improvement in beta cells functional activity. Furthermore, postbiotics are being explored as emerging treatment against diabetic microvascular complications like diabetic retinopathy.

Next, is the fecal microbiota transplantation (FMT), which may consider as a comprehensive microbiome-targeted therapy by transferring stool from a healthy donor into a T2DM recipient. Recent systematic review and meta-analysis evaluated the efficacy of FMT in T2DM patients found that FMT primarily impacts indices like HbA1c, fasting plasma glucose and insulin resistance [193]. Study reported that FMT from metabolically healthy donors can transiently improve peripheral insulin sensitivity, accompanied by shifts in gut microbiota composition and SCFA profiles [194, 195]. While multiple RCTs and animal studies show significant metabolic benefits, individual responses vary and data in established T2DM are limited. Therefore, it is crucial to conduct strict donor screening and standardized protocols for the safe use of FMT in the future. 

### Engineered Probiotics and Synthetic Biology Approaches

Next, the synthetic biology and next-generation probiotic technologies enable the engineering of *Lactobacillus* spp. with tailored functionalities for glycaemic control in T2DM for enhanced therapeutic applications. For example, CRISPR-based engineering of tailored probiotics allows precise genome editing and transcriptional regulation in these bacteria, aiming to enhance stability and biodelivery for targeted health interventions [196, 197]. For instance, the next-generation probiotic *L. plantarum*-pMG36e-GLP-1, expressing glucagon-like peptide-1 (GLP-1) demonstrated improvements in glycemic control, pancreatic function and liver metabolism in diabetic mouse models [198]. 

In a recent study, a genetically engineered strain of *Escherichia coli Nissle* 1917 (EcN)-GLP-1 demonstrated protective effects on the islet β-cells, reduced key inflammatory markers including TLR-4, p-NF-κB/NF-κB, and Bax/Bcl-2 besides helped restore balanced gut microbial diversity in diabetic mice models [199]. Overall, advancement of engineered bacterial therapies to produce therapeutic molecules or modulate the gut microbiota brings healthcare closer to personalized medicine by reducing patient outcomes and minimizing side effects.

## Conclusion

This review summarized the evidence from various studies, suggesting the potential therapeutic mechanisms of *Lactobacillus* spp. for T2DM treatment. These include modulation of glucose metabolism and transport, anti-inflammatory effects, anti-oxidative effects, and restructuring of the gut microbiota composition. Besides that, they may also exert its antidiabetic actions by enhancing functional foods, but the extent of the effects may vary. Despite promising findings, clinical translation is hindered by multiple challenges, including strain-specific survivability in the gastrointestinal tract, safety concerns and risk of antibiotic resistance gene transfer. Nevertheless, from the perspective of consumer-health, selected *Lactobacillus*-containing foods or supplements may offer supportive benefits but not replacement for established pharmacological therapy.

The future of *Lactobacillus*-based interventions in T2DM lies in adopting precision medicine frameworks. The integration of precision medicine, supported by multi-omics technologies and artificial intelligence enable personalized microbiome-based therapies. Personalized microbiome profiling and electronic health data can be leveraged to tailor probiotic strain selection, dosage and delivery methods, moving beyond the traditional "one-size-fits-all" model. Furthermore, advances in synthetic biology and next-generation probiotic engineering offer exciting opportunities to enhance the functionality of *Lactobacillus* strains. In a nutshell, a multidisciplinary approach with the combination of microbiology, genomics, artificial intelligence and clinical sciences are essential to unlock the full potential of targeted *Lactobacillus*-based therapies for personalized T2DM treatment.

## Key References

- Zhang C, Jiang J, Wang C, Li S, Yu L, Tian F, Zhao J, Zhang H, Chen W, Zhai Q. Meta-analysis of randomized controlled trials of the effects of probiotics on type 2 diabetes in adults. Clin Nutr. 2022 Feb;41(2):365–373. 10.1016/j.clnu.2021.11.037. Epub 2021 Dec 10. PMID: 34999331.
  - ○ This large-scale meta-analysis consolidates evidence from randomized controlled trials on probiotics in T2DM, including *Lactobacillus* strains, demonstrating their glycemic benefits.
- Zhao Q, Chen Y, Huang W, Zhou H, Zhang W. Drug-microbiota interactions: an emerging priority for precision medicine. Signal Transduct Target Ther. 2023 Oct 9;8(1):386. 10.1038/s41392-023-01619-w. PMID: 37806986; PMCID: PMC10560686.
  - ○ This article provides a cutting-edge overview of how gut microbiota influences drug response, directly supporting the rationale for pharmacomicrobiomics and potential of tailoring* Lactobacillus*-based interventions for T2DM treatment.
- Jiang S, Liu A, Ma W, Liu X, Luo P, Zhan M, Zhou X, Chen L, Zhang J. Lactobacillus gasseri CKCC1913 mediated modulation of the gut-liver axis alleviated insulin resistance and liver damage induced by type 2 diabetes. Food Funct. 2023 Sep 19;14(18):8504-8520. 10.1039/d3fo01701j. PMID: 37655696
  - ○ This article reported the strain-specific effects of *Lactobacillus *on the gut–liver axis, linking probiotic modulation to improved insulin resistance.

## Data Availability

No datasets were generated or analysed during the current study.
